# Severe Hemolysis during Primaquine Radical Cure of *Plasmodium vivax* Malaria: Two Systematic Reviews and Individual Patient Data Descriptive Analyses

**DOI:** 10.4269/ajtmh.23-0280

**Published:** 2023-08-21

**Authors:** Daniel Yilma, Emily S. Groves, Jose Diego Brito-Sousa, Wuelton M. Monteiro, Cindy Chu, Kamala Thriemer, Robert J. Commons, Marcus V. G. Lacerda, Ric N. Price, Nicholas M. Douglas

**Affiliations:** ^1^ Jimma University Clinical Trial Unit, Department of Internal Medicine, Jimma University, Jimma, Ethiopia;; ^2^ WorldWide Antimalarial Resistance Network, Oxford, United Kingdom;; ^3^ Division of Clinical Pharmacology, Department of Medicine, University of Cape Town, Cape Town, South Africa;; ^4^ Division of Global and Tropical Health, Menzies School of Health Research and Charles Darwin University, Darwin, Casuarina, Northern Territory, Australia;; ^5^ Instituto de Pesquisa Clínica Carlos Borborema, Fundacão de Medicina Tropical Dr. Heitor Vieira Dourado, Manaus, Brazil;; ^6^ Escola Superior de Ciências da Saude, Universidade do Estado do Amazonas, Manaus, Brazil;; ^7^ Shoklo Malaria Research Unit, Mahidol-Oxford Tropical Medical Research Unit, Faculty of Tropical Medicine, Mahidol University, MaeSot, Tak, Thailand;; ^8^ General and Subspecialty Medicine, Grampians Health, Ballarat, Victoria, Australia;; ^9^ Instituto Leônidas & Maria Deane, Fundacão Oswaldo Cruz, Manaus, Brazil;; ^10^ Centre for Tropical Medicine, Nuffield Department of Clinical Medicine, University of Oxford, United Kingdom;; ^11^ Mahidol-Oxford Tropical Medicine Research Unit, Faculty of Tropical Medicine, Mahidol University, Bangkok, Thailand;; ^12^ Department of Medicine, University of Otago, Christchurch, New Zealand;; ^13^ Department of Infectious Diseases, Christchurch Hospital, Christchurch, New Zealand

## Abstract

Primaquine (PQ) kills *Plasmodium vivax* hypnozoites but can cause severe hemolysis in patients with glucose-6-phosphate dehydrogenase (G6PD) deficiency. We conducted two systematic reviews. The first used data from clinical trials to determine the variety of definitions and frequency of hematological serious adverse events (SAEs) related to PQ treatment of vivax malaria. The second used data from prospective studies and case reports to describe the clinical presentation, management, and outcome of severe PQ-associated hemolysis necessitating hospitalization. In the first review, SAEs were reported in 70 of 249 clinical trials. There were 34 hematological SAEs among 9,824 patients with *P. vivax* malaria treated with PQ, nine of which necessitated hospitalization or blood transfusion. Criteria used to define SAEs were diverse. In the second review, 21 of 8,487 articles screened reported 163 patients hospitalized after PQ radical cure; 79.9% of whom (123 of 154) were prescribed PQ at ≥ 0.5 mg/kg/day. Overall, 101 patients were categorized as having probable or possible severe PQ-associated hemolysis, 96.8% of whom were G6PD deficient (< 30% activity). The first symptoms of hemolysis were reported primarily on day 2 or 3 (45.5%), and all patients were hospitalized within 7 days of PQ commencement. A total of 57.9% of patients (77 of 133) had blood transfusion. Seven patients (6.9%) with probable or possible hemolysis died. Even when G6PD testing is available, enhanced monitoring for hemolysis is warranted after PQ treatment. Clinical review within the first 5 days of treatment may facilitate early detection and management of hemolysis. More robust definitions of severe PQ-associated hemolysis are required.

## INTRODUCTION

*Plasmodium vivax* forms dormant liver stages (hypnozoites) that can reactivate periodically, causing recurrent blood-stage malaria infections known as relapses. Primaquine (PQ) is an 8-aminoquinoline drug that has been used for malaria treatment and prophylaxis since the 1950 s. It is the only widely available antimalarial that eliminates *P. vivax* hypnozoites and thus can prevent relapses. The WHO recommends PQ for radical cure of *P. vivax* or *Plasmodium ovale* malaria in all malaria transmission settings in children and adults except pregnant women, infants younger than 6 months, women breastfeeding infants younger than 6 months, and women breastfeeding older infants with unknown glucose-6-phosphate dehydrogenase (G6PD) status.^1^

Primaquine can cause severe drug-induced hemolysis, particularly in individuals predisposed to oxidant erythrocytic damage resulting from a functional deficiency of the enzyme G6PD.^2^^–^^4^ Glucose-6-phosphate dehydrogenase deficiency is the most common human enzymopathy globally and is particularly prevalent in malaria-endemic regions.^5^^,^^6^ Patients with PQ-induced hemolysis can develop severe anemia necessitating blood transfusion and hemoglobinuria, potentially leading to kidney damage.^7^ If severe, PQ-associated hemolysis can be fatal.^8^

The WHO recommends G6PD activity assessment prior to administration of PQ for radical cure,^1^ but there is limited access to practicable assays in malaria-endemic regions.^9^ Primaquine treatment is therefore often not implemented because of the risks of hemolysis with unguided use.^10^ Conversely, patients with *P. vivax* malaria who are not treated with PQ radical cure can have major deleterious consequences from multiple relapses, including severe anemia.^11^^,^^12^ Policymakers need to weigh the potential risks of PQ-associated hemolysis against the risks of recurrent malaria when deciding on treatment protocols. To do this, they need robust data on the incidence and outcomes of PQ-attributable severe hemolysis.

The diagnosis of hemolysis secondary to PQ is typically based on a decrease in hemoglobin (Hb) and/or clinical manifestations suggestive of intravascular hemolysis. Definitions for severe or clinically significant PQ-associated hemolysis are not established. Attribution of hemolysis to PQ is difficult in the presence of concomitant parasitemia.

We conducted two complementary systematic reviews to inform and standardize PQ pharmacovigilance practices and to facilitate quantification of the risks and benefits of different radical cure strategies. The aim of the first review was to determine the variety of definitions and frequency of hemolytic serious adverse events (SAEs) in patients enrolled in prospective *P. vivax* antimalarial clinical efficacy trials. The second review used individual patient data from case reports and prospective studies to describe the clinical presentation, management, and outcome of patients with severe PQ-associated hemolysis who required hospitalization for clinical management.

## MATERIALS AND METHODS

### Review 1: SAEs in clinical trials.

The first systematic review used data from the Worldwide Antimalarial Resistance Network (WWARN) “vivax surveyor.”^13^ This repository contains details of all *P. vivax* antimalarial clinical efficacy studies done between January 1960 and August 23, 2021 (registered previously at PROSPERO [CRD42016053228]). To be eligible for inclusion in the current review, trials had to have been done since 1990 and include one or more treatment arms in which patients were treated with either partial or fully supervised PQ therapy for at least 5 days (Supplemental Appendix S1, Box S1). Primaquine administration had to commence within 7 days of starting blood schizontocidal therapy. Data from non-PQ–containing arms in these studies were also extracted for comparative purposes. Studies meeting these criteria, but not reporting the presence or absence of adverse effects of treatment, were excluded.

Generic definitions of SAEs have been standardized^14^ and, generally speaking, encompass events that are life-threatening or require hospitalization. In this review, the respective authors’ classification of events as SAEs was accepted, but the clinical and laboratory criteria used by the authors to classify the events as SAEs were outcomes of interest.

Data were extracted into Microsoft Excel (Microsoft Corp., Redmond, WA, USA), including the number of patients enrolled in each treatment arm, patient demographics, clinical and laboratory features, the number of patients experiencing any author-reported SAE, the relationship of SAEs to PQ administration, and the number of hematological SAEs. Severe hematological adverse events (defined a priori as a hematological event requiring hospitalization, blood transfusion, or renal replacement therapy, or death) were assigned after data extraction based on the clinical data available. Original investigators were approached for missing data and, when possible, the clinical and laboratory data used by the original investigators to classify episodes as hematological SAEs were collected for individual patients. Laboratory methods used to screen for G6PD deficiency (if performed) and individual G6PD activity measurements of patients experiencing a hematological SAE were also collected.

### Review 2: severe PQ-associated hemolysis.

In the second systematic review, all articles in any language reporting at least one case of severe PQ-associated hemolysis were identified. To be eligible for inclusion, works had to report data attributable to individual patients receiving PQ for *P. vivax* radical cure or terminal prophylaxis. The review was registered at PROSPERO (CRD42020196604). The term severe hemolysis was used to define a clinical event requiring hospitalization, blood transfusion, or renal replacement therapy, or death, which the original investigators attributed to hemolysis or anemia. Children younger than 6 months of age, and pregnant or lactating women were excluded from the analysis as both are ineligible for PQ treatment.

We searched PubMed, Web of Science, Embase, and the Cochrane Central Database for eligible reports published between January 1, 1940 and May 20, 2020, according to PRISMA guidelines (Supplemental Appendix S1, PRIMSA Checklist). The search terms are presented in (Supplemental Appendix S1, Box S2). We also searched articles included in the WWARN *P. vivax* clinical trial database used in the first systematic review,^13^ as well as reference lists from identified articles and documents, conference abstracts, and unpublished works from our network of collaborators. The review process was undertaken by six independent reviewers (D. Y., E. G., K. T., R. J. C., N. M. D., and R. N. P.), with discrepancies resolved by discussion.

Data from relevant reports were extracted into a RedCap database (hosted at Charles Darwin University), including the source of data, patient demographics, PQ dose regimen, G6PD activity, and all available clinical and laboratory features of the hemolytic event (see Supplemental Appendix S2). Authors of eligible studies were contacted and asked to provide relevant additional information that was not reported in the publication. All data were de-identified. From the data available, the certainty of the diagnosis of PQ-associated severe hemolysis was categorized as probable, possible, or uncertain using an a priori hierarchical evidence scale (Table 1).

**Table 1:** Classification of certainty of the diagnosis of primaquine-associated severe hemolysis

Probable
Paired Hb* recorded
Decrease in Hb to < 7 g/dL and > 25% fractional decrease
Decrease in Hb to ≥ 5 g/dL
Decrease in Hb to 3 to 4.99 g/dL or > 25% decrease AND dark-brown or red urine or jaundice or shortness of breath or heart failure or blood transfusion or renal replacement therapy or death
Single Hb† recorded after PQ administration
Hb < 7 g/dL AND dark, brown or red urine or jaundice AND shortness of breath or heart failure or blood transfusion or renal replacement therapy or death
Possible
No Hb recorded
Dark urine or jaundice AND blood transfusion or renal replacement therapy or death
Single Hb recorded after PQ administration
Hb < 7 g/dL AND dark, brown, or red urine or jaundice or shortness of breath or heart failure or blood transfusion or renal replacement therapy or death
Hb ≥ 7 g/dL AND dark urine or jaundice AND shortness of breath or heart failure or blood transfusion or renal replacement therapy or death
Uncertain
Not fulfilling probable or possible criteria but considered serious PQ-associated hemolysis by authors

Hb = hemoglobin; PQ = primaquine.

* Hemoglobin reported before and after PQ administration.

† Hemoglobin reported only after PQ administration.

All cases required temporal association with PQ within the preceding 4 weeks.

### Statistical analyses.

Statistical analyses and graphing were conducted using STATA version 15.1 (StataCorp, College Station, TX, USA). The dose of PQ was categorized according to the daily dose as low (< 0.375 mg/kg/day), intermediate (0.375 < 0.75 mg/kg/day), or high (≥ 0.75 mg/kg/day). Continuous variables were presented as median, range, and interquartile range (IQR). Categorical variables were presented as number and proportion. Qualitative G6PD testing was assumed to detect deficiency in patients with less than 30% enzyme activity. The risks of SAEs reported in clinical trials were stratified a priori into three groups: patients with unknown G6PD status, patients with G6PD activity > 30%, and females with G6PD activity > 30%.

## RESULTS

### First review: SAEs reported in clinical trials.

**Figure 1.**
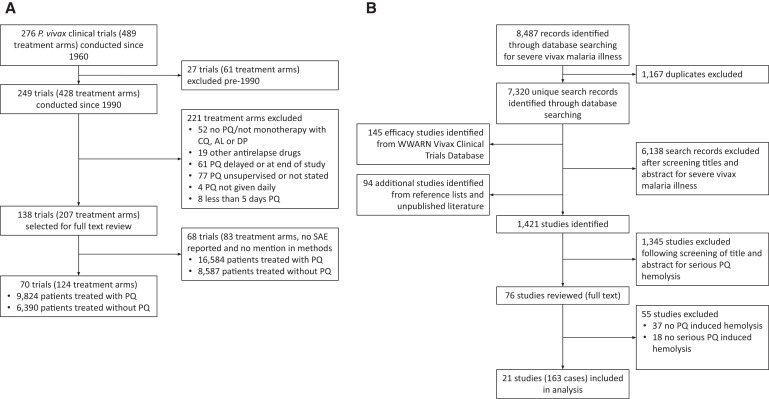
Data extraction process for (**A**) review of serious adverse effects (SAEs) in clinical trials and (**B**) review of severe primaquine-associated hemolysis. AL = artemether-lumefantrine; CQ = chloroquine; PQ = primaquine; WWARN = Worldwide Antimalarial Resistance Network.

Between January 1990 and August 2021, 249 published *P. vivax* clinical trials (428 treatment arms) were identified, of which 138 (55.4%) fulfilled the inclusion criteria and were reviewed for SAEs (Figure 1). Seventy trials (124 treatment arms, 16,214 patients; *n *= 44 randomized studies [62.9%]) reported adverse events explicitly and included collectively 9,824 patients treated with PQ. A further 6,390 patients were not treated with PQ (Table 2).

**Table 2:** Risks of serious adverse events reported in clinical trials

Serious adverse event	Total (*n*/*N*)	No PQ		Low daily dose PQ (< 0.375 mg/kg/day)		Intermediate daily dose PQ (0.375 to < 0.75 mg/kg/day)		High daily dose PQ (≥ 0.75 mg/kg/day)	
		*n*/*N*	Rate per 100,000 (95% CI)	*n*/*N*	Rate per 100,000 (95% CI)	*n*/*N*	Rate per 100,000 (95% CI)	*n*/*N*	Rate per 100,000 (95% CI)
Any SAE									
No G6PD testing	24/5,707	1/3,599	28 [1–155]	9/1,048	859 (393–1,624)	14/1,060	1,321 (724–2,206)	–	–
G6PD testing > 30%	83/10,056	7/2,604*	269 [108–554]	12/2,658†	452 (234–787)	31/3,466	894 (609–1,267)	33/1,328	2,485 (1,717–3,472)
Any PQ-related SAE									
No G6PD testing	22/5,707	1/3,599	28 [1–154]	8/1,048	763 (330–1,499)	13/1,060	1,226 (655–2,088)	–	–
G6PD testing > 30%	50/10,056	3/2604*	115 [24–336]	11/2,658†	414 (207–739)	16/3,466	462 (264–749)	20/1,328	1,506 (922–2,316)
Hemolytic SAE									
No G6PD testing	17/5,707	0/3,599	0	5/1,048	477 (155–1,110)	12/1,060	1,132 (586–1,969)	–	–
G6PD testing > 30%	17/10,056	3/2,604*	115 [24–336]	8/2,658†	301 (130–592)	1/3,466	29 (1–161)	5/1,328	376 (122–876)
Females > 30%	9/2,782	0/632	0	4/757†	661 (215–1,535)	0/876	0	5/517	967 (315–2,242)
Severe hemolytic SAE‡									
No G6PD testing	0/5,707	0/3,599	0	0/1,048	0	0/1,060	0	–	–
G6PD testing > 30%	8/10,056	0/2,604*	0	5/2658†§	188 (61–438)	0/3,466	0	3/1,328‖	226 (47–660)
Females > 30%	7/2,782	0/632	0	4/757†§	661 (215–1,535)	0/876	0	3/517‖	580 (120–1,696)

CI = confidence interval; G6PD = glucose-6-phosphate dehydrogenase; PQ = primaquine; SAE = serious adverse event.

* Excludes 187 patients tested at > 70% activity.

† Excludes 264 patients tested at > 70% activity.

‡ Fulfilling criteria of hemolysis requiring hospitalization, blood transfusion, renal replacement therapy, or death.

§ All five cases (four females) came from one study and were hospitalized with jaundice and dark urine, but minor decreases in hemoglobin (0.6–2.0 g/dL).

‖ Includes three heterozygous females, two requiring blood transfusion.

Numerators exclude patients with G6PD deficiency (≤ 30%) enrolled and treated in error. Denominators excluded 68 trials (16,584 patients treated with PQ and 8,587 without PQ) in which no SAEs were reported, and in which no mention was made of reporting of SAEs in methods or results. Raw data are presented in review1b and review1c sheets in Supplemental Appendix S2.

A total of 110 SAEs were reported, of which 68 (67.3%) were attributed by the authors to PQ treatment. There were 37 episodes of acute hemolysis; 34 occurred in patients treated with PQ and three occurred in patients treated without PQ. Three hemolytic SAEs occurred in patients with G6PD activity < 30% who had been enrolled and treated with PQ in error, two of whom met the criteria for inclusion in our second systematic review (both required blood transfusion and one was hospitalized).

The reasons stated for classification as a hemolytic SAE included 16 patients who had a > 2.5g/dL decrease in Hb or a > 25% decrease (in all cases PQ was continued without interruption), five patients who had a > 3g/dL decrease in Hb or a > 30% decrease (PQ was continued in four cases), three patients who had dark urine, and six patients who had a decrease in Hb to < 7 g/dL (three required blood transfusion and, in two of the remaining cases, PQ was ceased temporarily and restarted). A further six patients, all in the same study, were hospitalized for management, although their Hb nadir ranged from 8.5 to 13.2 g/dL, with a maximum absolute decrease in Hb of 2 g/dL. One patient was reported as having acute hemolysis with jaundice (total bilirubin, 105 μmol/L), but no further details could be ascertained. Overall, 27.2% (10 of 37) of the reported hemolytic SAEs were classified by us as severe based on the need for hospitalization, blood transfusion, renal replacement therapy, or death.

The risks of SAEs and hemolysis in patients with unknown G6PD status or G6PD activity ≥ 30% are presented in Table 2. The overall incidence of PQ-associated hemolytic SAEs (all PQ dosing regimens) was 188/100,000 (95% confidence interval [CI], 103–315), and the overall risk of severe PQ hemolysis was 107/100,000 (95% CI, 46–212).

### Second review: reports of severe hemolysis.

In the second review, 8,487 articles were identified describing patients with vivax malaria requiring admission to hospital. After removing 1,167 duplicate publications, adding a further 239 articles (identified from clinical trials and previous reviews), and excluding articles after screening of the titles and abstracts, 76 articles remained for full-text review (Figure 1). Fifty-five studies were excluded subsequently because they did not report patients fulfilling the criteria for severe PQ-associated hemolysis. A total of 163 individuals with severe PQ-associated hemolysis were identified from 21 articles published between 1953 and 2020: 43 were from Asia (13 articles), 116 from South America (5 articles), 3 from North America (2 articles), and 1 from Oceania (1 article) (Figure 1). Seventeen patients (8 articles) were reported in randomized controlled trials and 146 patients (13 articles) were reported in case reports. A summary of the studies included in the analysis is provided in Supplemental Appendix S2.

The median age of individuals with severe PQ-associated hemolysis was 18 years (IQR, 12–28; range, 1–84) and 132 (80.9%) were male. Overall, 134 patients (82.2%) had *P. vivax* infection and six (3.7%) had mixed *P. vivax* and *P. falciparum* infection. A further 23 individuals (14.1%) were negative when tested for malaria, 19 of whom were presumed by the authors to have had previous *P. vivax* infection. Of the 140 individuals treated for acute malaria, 135 (96.4%) also received chloroquine, 4 (2.9%) received dihydroartemisinin-piperaquine, and 1 (0.7%) received amodiaquine.

The duration of the PQ regimen prescribed was reported in 156 cases. Eighteen patients (11.5%) were prescribed 14 days of treatment; 1 patient (0.6%), 8 days of treatment; 121 patients (77.6%), 7 days of treatment; and 16 patients (10.3%), 5 days or less. Two patients (1.3%) were prescribed weekly PQ administration for 8 weeks. The target daily PQ doses prescribed are provided in Table 3. The total dose prescribed was < 2.5 mg/kg for 16 patients (10.3%), 2.5 to 5 mg/kg for 132 patients (84.6%), and ≥ 5 mg/kg for 8 patients (5.1%).

**Table 3:** Characteristics and clinical outcomes of individuals with severe primaquine-associated hemolysis

Clinical manifestations of severe hemolysis	Probable PQ associated (*n* = 48)		Possible PQ associated (*n *= 53)		Uncertain PQ associated (*n* = 62)	
	*N*	*n* (%)	*N*	*n* (%)	*N*	*n* (%)
Symptoms and signs						
Dark urine	36	29 (80.6)	38	34 (89.5)	39	38 (97.4)
Pallor	40	38 (95.0)	35	31 (88.6)	40	36 (90.0)
Dyspnea	33	9 (27.3)	26	7 (26.9)	30	2 (6.7)
Dizziness	17	9 (52.9)	9	4 (44.4)	8	3 (37.5)
Fever	38	24 (63.2)	31	16 (51.6)	32	7 (21.9)
Severe fatigue	20	13 (65.0)	6	5 (83.3)	3	2 (66.7)
Heart failure	31	3 (9.7)	31	6 (19.4)	33	0 (0.0)
Chest pain	7	1 (14.3)	3	0 (0.0)	0	0 (0.0)
Jaundice	44	39 (88.6)	48	44 (91.7)	54	47 (87)
Low urine output	14	2 (14.3)	4	0 (0.0)	5	2 (40)
Laboratory findings						
Glucose 6-phosphate dehydrogenase						
Normal	47	0 (0.0)	51	1 (2.0)	61	5 (8.2)
Intermediate	47	2 (4.4)	51	0 (0.0)	61	0 (0.0)
Deficient	47	45 (95.7)	51	50 (98.0)	61	56 (91.8)
Total bilirubin, > 1 mg/dL	37	31 (83.8)	38	35 (92.1)	52	46 (88.5)
Indirect bilirubin, > 0.8 mg/dL	36	30 (83.3)	36	33 (91.7)	43	37 (86.1)
Urine dipstick blood, positive	21	10 (47.6)	27	19 (60.4)	31	21 (67.7)
Urine dipstick urobilinogen, positive	20	3 (15)	25	14 (66)	30	12 (40)
White blood cell count, > 12,000/μL	32	20 (62.5)	38	20 (52.6)	36	10 (27.8)
Target daily dose of PQ						
0.25 mg/kg/day	47	5 (10.6)	52	12 (23.1)	57	14 (24.6)
0.5 mg/kg/day	47	36 (76.6)	52	39 (75.0)	57	43 (75.4)
0.75 mg/kg/day	47	2 (4.3)	52	0 (0.0)	57	0 (0.0)
1.0 mg/kg/day	47	4 (8.5)	52	1 (1.9)	57	0 (0.0)
Management						
Blood transfusion	48	45 (93.8)	42	31 (73.8)	43	0 (0.0)
Renal replacement therapy	39	4 (10.3)	41	4 (9.8)	42	0 (0.0)
Hospitalized (no blood transfusion or renal replacement therapy)	48	3 (6.3)	41	8 (19.5)	42	42 (100)
Outcome						
Died	48	0 (0.0)	53	7 (13.2)	62	0 (0.0)

*N* = number of patients for whom data were available; *n* = number of patients with manifestation present.

Glucose-6-phosphate dehydrogenase status was recorded for 159 of the 163 patients (97.5%), with a qualitative assay used in 145 patients (91.2%) and a quantitative assay in 10 patients (6.3%). The assay method was not stated in four patients (2.5%). Overall, 150 individuals (94.3%) were categorized as G6PD deficient and two (1.3%) had intermediate G6PD deficiency. Seven patients (4.4%) were G6PD normal according to a qualitative G6PD test (assumed to have ≥ 30% activity), four of whom were female. The certainty of the diagnosis of severe PQ-associated hemolysis was categorized as probable in 48 patients (29.5%), possible in 53 patients (32.5%), and uncertain in 62 patients (38.0%). Glucose-6-phosphate dehydrogenase activity was documented in 98 of the patients with probable or possible severe hemolysis, of whom 96.9% (*n* = 95) were G6PD deficient (< 30% activity) and three were heterozygous females (64%, 94%, and > 30% enzyme activity, respectively).

### Clinical presentation and management of patients.

All subsequent results are restricted to the 101 patients with probable or possible severe PQ-related hemolysis, 95 of whom were G6PD deficient (<30%). Three patients were heterozygous females with >30% activity and three patients did not have a reported G6PD status. The most common clinical presentations of hemolysis were pallor (92.0%, 69 of 75), dark urine (85.1%, 63 of 74), and jaundice (90.2%, 83 of 92) (Tables 3 and 4).

**Table 4:** Clinical and laboratory manifestations of patients with probable or possible severe primaquine-associated hemolysis by clinical outcome

Clinical or laboratory manifestation	Blood transfusion (*N* = 76)		Renal replacement therapy (*N* = 8)		Death (*N* = 7)		All patients (*N* = 101)	
	*N*	*n* (%)	*N*	*n* (%)	*N*	*n* (%)	*N*	*n* (%)
Clinical manifestations								
Dark urine	57	48 (84.2)	5	5 (100)	4	4 (100)	74	63 (85.1)
Pallor	60	56 (93.3)	8	7 (87.5)	2	2 (100)	75	69 (92.0)
Dyspnea	47	8 (17.0)	7	2 (28.6)	3	3 (100)	59	16 (27.1)
Dizziness	22	11 (50.0)	4	1 (25.0)	0	0 (0.0)	26	13 (50.0)
Fever	58	37 (63.8)	7	4 (57.1)	1	1 (100)	69	40 (57.8)
Severe fatigue	21	15 (71.4)	0	0 (0.0)	0	0 (0.0)	26	18 (69.2)
Heart failure	51	7 (13.7)	6	6 (100)	0	0 (0.0)	62	9 (14.5)
Chest pain	8	1 (12.5)	8	0 (0.0)	0	0 (0.0)	10	1 (10.0)
Jaundice	67	59 (88.1)	7	7 (100)	6	6 (100)	92	83 (90.2)
Low urine output	14	2 (14.3)	3	2 (66.7)	0	0 (0.0)	18	2 (11.1)
Laboratory findings								
Hemoglobin > 3 g/dL decrease	11	9 (81.8)	0	0 (0.0)	0	0 (0.0)	14	12 (85.7)
Hemoglobin > 25% decrease	11	11 (100)	0	0 (0.0)	0	0 (0.0)	14	14 (100)
Total bilirubin > 1 mg/dL	59	51 (86.4)	7	5 (71.4)	1	1 (100)	75	66 (88.0)
Indirect bilirubin > 0.8 mg/dL	59	51 (86.4)	7	5 (71.4)	1	1 (100)	72	63 (87.5)
Urine dipstick blood, positive	31	17 (54.8)	6	3 (50.0)	5	5 (100)	48	29 (60.4)
Urobilinogen dipstick, positive	30	5 (16.7)	5	1 (20.0)	3	3 (100)	45	17 (37.8)
White blood cell count > 12,000/μL	56	33 (58.9)	7	4 (57.1)	3	3 (100)	70	40 (57.1)

*N* = number of patients for whom data were available, *n* = number of patients with manifestation present.

Patients could present with more than one outcome. Thus, the total number of patients does not equal the summation of those who received a blood transfusion and/or renal replacement therapy, or who died.

Fourteen patients had an Hb measurement before and after initiation of PQ. In these patients, the median decrease in Hb was 4.4 g/dL (IQR, 3.7–6.6; range, 2.2–8.9). Of the patients in whom serum bilirubin was measured, 88% (66 of 75) had an increased total bilirubin concentration (median, 4 mg/dL; IQR, 2.1–5.5; range, 0.3–46.4). At the time that patients were hospitalized, 79.5% (58 of 73) of those with acute malaria were aparasitemic; however, 55.3% (26 of 47) were febrile and 62.5% (30 of 48) had an increased total white cell count.

### Timing of severe PQ-associated hemolysis.

**Figure 2.**
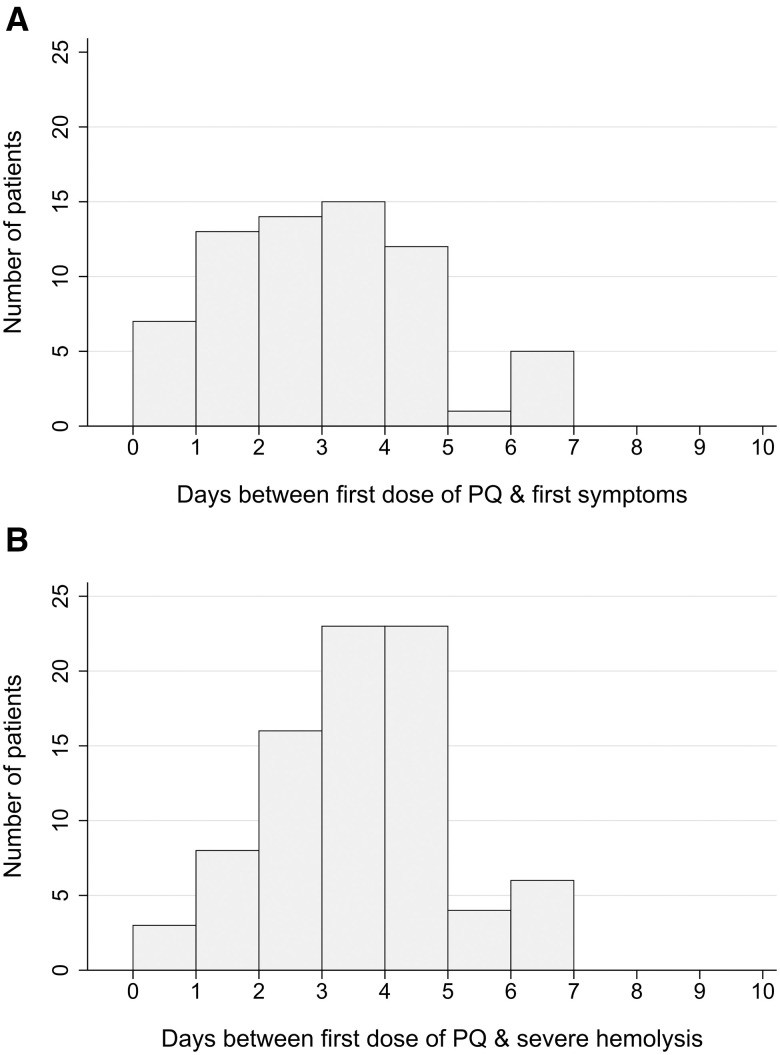
Timing of the first symptoms of hemolysis (**A**) and onset of severe hemolysis (**B**) after commencing primaquine (PQ).

**Figure 3.**
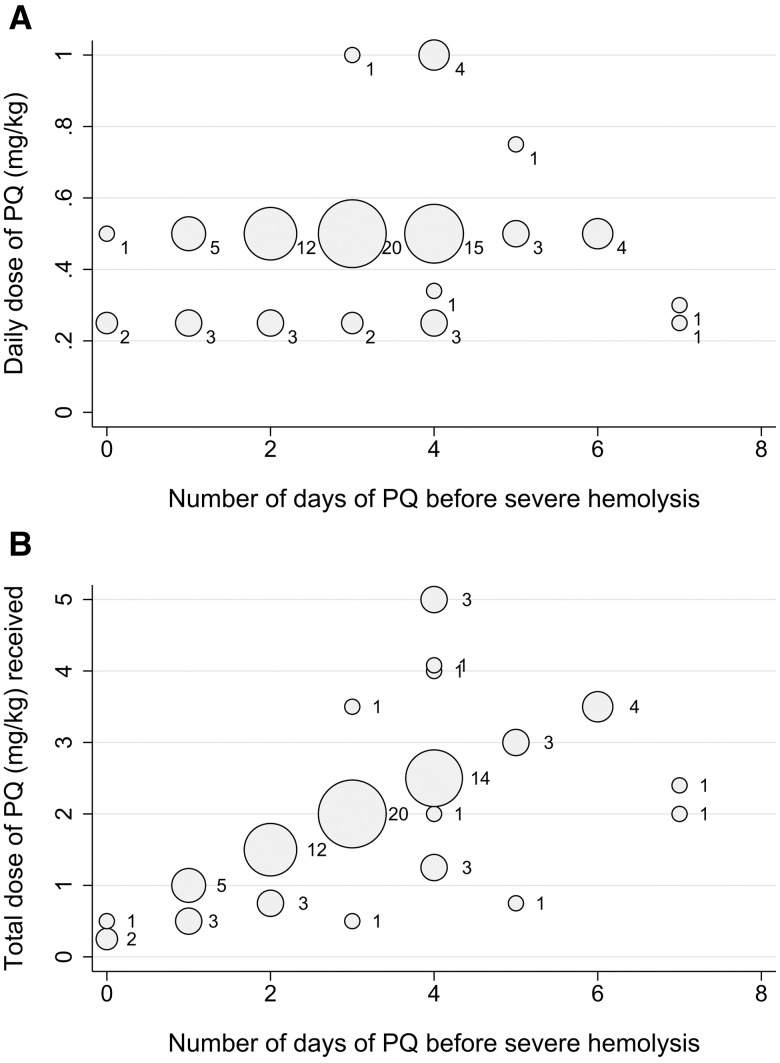
Timing of hospital admission with severe primaquine (PQ)-associated hemolysis according to the daily dose of PQ (**A**) and total PQ dose administered at that time (**B**). The size and label of each dot in the figure indicates the number of events. Data are restricted to 101 probable and possible cases of severe PQ-associated hemolysis.

Information on the timing of manifestations of severe PQ-associated hemolysis was available for 93 patients (92.1%), with all patients presenting within the first 7 days of commencing PQ treatment. Overall, 89.2% (83 of 93) developed symptoms of hemolysis within 4 days of commencing PQ. The first symptoms of hemolysis were reported primarily on day 2 or 3 (43.3%) after commencing PQ, whereas onset of severe hemolysis occurred most frequently on day 3 or 4 (55.4%) (Figure 2). At the onset of manifestations of severe hemolysis, PQ had been taken for a median of 4 days (range, 1–8), with a median total dose received of 2.0 mg/kg (IQR, 1.5–2.5; range, 0.3–5.0) (Supplemental Appendix S1, Figure S1). In total, 82.8% (82 of 99) of the cases of probable or possible PQ-associated severe hemolysis occurred after administration of a daily dose ≥ 0.5 mg/kg (Figure 3).

### Outcomes.

**Figure 4.**
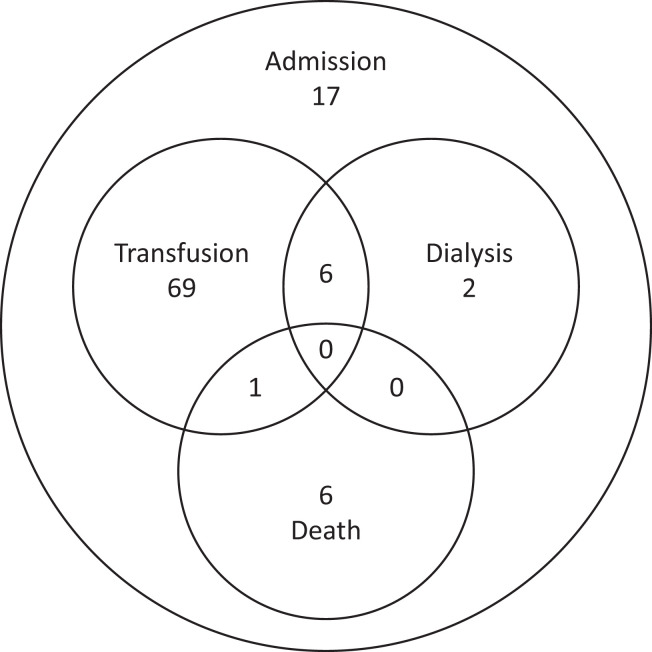
Outcomes following severe primaquine (PQ)-associated hemolysis. Data are restricted to 101 probable and possible cases of severe PQ-associated hemolysis.

Seventy-six of 90 patients (83.5%) with severe PQ-associated hemolysis required blood transfusion and 10.0% (8 of 80) required renal replacement therapy (Figure 4). The absolute decrease in Hb could not be calculated for any of the patients requiring renal replacement therapy. Seven of the 101 patients (6.9%) with severe PQ-associated hemolysis died. All but one of the deaths occurred in males. Four deaths were reported in Sri Lankan children age 1, 2, 3, and 12 years old, all of whom received PQ for radical cure of *P. vivax.* The cause of death was reported as cardiac failure from anemia in three of these patients and irreversible renal failure in one. Three deaths were reported in Brazilian patients, all males age 13, 33, and 57 years. Overall, five of the seven patients who died were reported to be G6PD deficient, with the G6PD status unknown in the remaining two cases. One of the patients who died received a blood transfusion, but information on blood transfusion or renal replacement therapy was not documented for the remaining six patients. Of the patients who survived, 72.7% (56 of 77) were discharged from the hospital within 1 week of admission.

## DISCUSSION

Radical cure of *P. vivax* malaria requires treatment of both blood and liver stages of the parasite to ensure clinical recovery and reduce the risk of subsequent relapse. Without radical cure, relapses occur in up to 70% of patients, the risk and frequency of which vary with host immunity, background transmission intensity, and geographic location.^15^ The antirelapse efficacy of PQ is related to the total dose of PQ administered, whereas for the 7- and 14-day regimens, the risk of hemolysis is related to the daily dose.^16^^,^^17^ Because routine G6PD testing is often unavailable in malaria-endemic countries, most national malaria control programs currently recommend a total dose of 3.5 mg/kg administered over 14 days, allowing the daily dose to be reduced to 0.25 mg/kg to minimize hemolysis.

Adherence to standard 14-day PQ regimens is typically poor.^18^ Seven-day regimens of higher daily doses of PQ (0.5 mg/kg) are used routinely in several endemic countries, including Brazil, Peru, and China,^10^ providing a total dose of 3.5 mg/kg. Recent evidence suggests that 7 mg/kg may be required to reduce the risk of recurrent parasitemia to less than 10% in areas of both high and low relapse periodicity.^19^^,^^20^ Giving 7 mg/kg over 7 days as opposed to 14 days (1 mg/kg/day) is equally efficacious, but the increased daily dose is associated with a greater risk of SAEs.^21^^,^^22^

Thirty-seven hemolytic SAEs were reported in the clinical trials in our first review, 34 of which occurred after PQ administration. The criteria used to classify these adverse events as serious varied. Twenty-one reported SAEs were based solely on a significant decrease in Hb (> 2–3 g/dL), with all but one of these patients continuing their course of PQ to completion. In total, 10 of the patients with reported SAEs required hospitalization and blood transfusion for medical management. The overall risk of severe PQ hemolysis was estimated to be 107/100,000 patients with normal G6PD activity (≥ 30% activity) treated with PQ.

The prospective clinical trials in our reviews generally applied more conservative criteria for classifying adverse events as serious (presumably with the aim of ensuring patient safety in a controlled environment) than the case reports and pharmacovigilance studies in our second review. The latter often had limited clinical and laboratory information available prior to the onset of the events. Even when comprehensive data are available, the clinical and laboratory features that justify hemolysis being classified as serious or life-threatening have yet to be defined. This results in a lack of consistent data for decisionmakers to balance the risks of PQ radical cure with the risks of multiple relapses of vivax malaria in different endemic locations.^1^ Quantifying these risks will require robust definitions of hemolysis that can accommodate differential access to laboratory investigations. Standardized templates for reporting relevant clinical and laboratory details of potential hemolytic events will facilitate this process greatly. An example of a form for reporting the key data identified in our analysis is provided in Supplemental Appendix S1.

The second of our systematic reviews included all articles published since the introduction of PQ. The largest case series were reported from South America, where the prevalence of G6PD deficiency is high, and comparatively large daily doses (0.5 mg/kg) of PQ are recommended.^8^^,^^23^ Conversely, no severe hemolytic events were reported from Africa, where G6PD deficiency is mostly attributable to the A− variant, which is associated with a lower risk of PQ-induced hemolysis.^5^ Ascertainment of severe hemolysis cases from Africa is likely to have been lower than in South America because commonly used rapid diagnostic tests are generally *P. falciparum* specific and therefore do not detect cases of vivax malaria, and PQ is rarely prescribed for radical cure of *P. vivax* or *P. ovale* malaria.^10^ Based on a priori criteria for certainty of association, 101 cases of severe hemolysis were categorized as probably or possibly related to PQ, all but one of which occurred in patients with either intermediate or severe G6PD deficiency. The first signs and symptoms of hemolysis occurred within 4 days of starting treatment in more than 90% of cases, with hospitalization occurring most frequently on day 4 after a median total dose of 2 mg/kg. All patients presented to hospital within the first week after starting PQ. Most cases occurred in patients receiving ≥ 0.5 mg/kg/day. The signs and symptoms of severe PQ-associated hemolysis in our review concurred broadly with those found in G6PD-deficient patients treated with the sulfur-based antimalarial drug dapsone.^24^

Twelve deaths probably or possibly related to PQ-associated hemolysis have been reported in the scientific literature, of which seven followed PQ for radical cure of *P. vivax* and were included in our second review.^4^ Although other deaths have undoubtedly occurred and gone unreported, this outcome is rare, with estimates varying from 1 in 625,000 to 1 in 110,000 patients treated with PQ.^4^^,^^8^ Of the seven patients in our analysis who died, five had a recorded G6PD status and were deficient at the 30% threshold.^8^^,^^25^^,^^26^ Of the 101 patients with probable or possible severe hemolysis, 98 had G6PD status recorded and all but three of these patients had G6PD deficiency with activity less than 30%. The remaining three patients were heterozygous females treated with high-dose PQ (1 mg/kg/day).^21^ Although there is an increased risk of hemolysis in heterozygous females with intermediate deficiency (30–70%),^27^ our findings suggest that for low (0.25 mg/kg) and intermediate (0.5 mg/kg) daily doses, severe hemolysis can be minimized by a reliable qualitative test used to exclude exposure of individuals with less than 30% enzyme activity.

Widespread point-of-care testing for G6PD deficiency prior to PQ administration will facilitate exclusion of patients at greatest risk of hemolysis and reduce adverse drug reactions. However, even in optimal conditions, G6PD testing is not 100% reliable and, as a result of logistical, financial, and supply constraints, quality tests may not always be available. In these cases, the risks of PQ-associated hemolysis need to be balanced against the cumulative risks of recurrent episodes of parasitemia and associated morbidity and mortality.^1^^,^^28^ In this context, it is critical to understand the clinical presentation and outcomes of severe PQ-associated hemolysis so that patients with impending hemolysis can be identified, and mitigating strategies enacted to avoid further deterioration.

Primaquine-associated hemolysis is most prominent during the first week of treatment, as circulating senescent erythrocytes with low G6PD activity and the greatest vulnerability to oxidant stress are cleared.^27^ With time, increased production of relatively G6PD-replete reticulocytes compensates in part for the increased red cell loss and increases the median tolerance of circulating erythrocytes to oxidative stress.^29^ This presumably explains the lack of new presentations with severe hemolysis beyond 1 week of starting PQ treatment. Hospitalization was delayed with respect to symptom onset by 1 to 2 days, suggesting there is an opportunity to prevent severe manifestations of hemolysis with early intervention. Based on these observations, routine clinical review within 5 days of starting PQ would identify most patients at risk of severe hemolysis and would allow early cessation of PQ to prevent further clinical deterioration.^30^ Tafenoquine is a novel 8-aminoquinoline that has a very long terminal elimination half-life and therefore can be given as a single dose to prevent *P. vivax* relapse. It has a similar hemolytic potential to PQ, and drug exposure cannot be stopped should severe hemolysis occur. Its use is therefore restricted to patients at low risk of hemolysis with ≥ 70% G6PD activity.

The strengths of our review include the use of consistent and strict a priori definitions for severe hemolysis and categorization of likelihood of a causal association with PQ. Individualized patient data were used and authors approached for additional unpublished information. Despite this, clinical information available for many patients was insufficient to enable a full assessment of likely causality. Our assessment of the frequency of severe PQ-associated hemolysis was subject to ascertainment bias, because many cases were not reported formally in the medical literature. Conversely, despite our attempts to define a causal association with drug administration, some hemolytic events may have been attributed inadvertently to PQ. A previous pooled analysis has shown that approximately 1 in 1,000 patients with *P. vivax* malaria treated without PQ had acute decreases in Hb (> 5 g/dL),^17^ which is remarkably similar to the risk of severe hemolysis we estimated in patients with > 30% enzyme activity (107/100,000). In our series, four patients who were G6PD deficient developed severe hemolysis on the day of initial presentation within hours of receiving the first dose of PQ. It is likely that these cases were attributable to acute malaria rather than drug administration.

In conclusion, severe PQ-associated hemolysis with clinical decompensation requiring urgent medical intervention is rare, but when it does occur, the first signs tend to become apparent within 2 to 3 days of starting treatment. Routine clinical review within 5 days to screen for symptoms such as dark urine, jaundice, pallor, severe fatigue, and fever would facilitate early detection of the majority of cases of impending severe hemolysis, enabling remedial action to be taken to prevent adverse outcomes. Diverse criteria have been used to categorize hematological SAEs, and this limits comparisons of their frequency among different locations and dosing regimens. Standardized definitions would facilitate valid pharmacovigilance in clinical trials and real-world settings. Although widely available point-of-care testing for G6PD deficiency is critical for reducing the risk of severe adverse drug reactions in vulnerable patients, severe hemolysis may still occur as a result of parasite-induced hemolysis, idiosyncratic reactions, and diagnostic errors. Hence, any public health intervention must include community engagement to ensure patients and health-care providers are aware of the hemolytic risks of malaria and its treatment, and the need to re-present for medical review in case of clinical deterioration.

## Supplemental Materials


Supplemental materials

Supplemental materials
